# Imagining the Future: Future Imagination Training Decreases Delay Discounting Among Internet Addicts and Non-Problematic Users

**DOI:** 10.3389/fpsyg.2021.731708

**Published:** 2021-10-21

**Authors:** Hongxia Li

**Affiliations:** School of Labor Economics, Capital University of Economics and Business, Beijing, China

**Keywords:** intertemporal choice, future imagination training, delay discounting rate, Internet addiction, future thinking

## Abstract

To test whether future imagination can reduce the delay discounting rate of the Internet addicts, we recruited 40 Internet addicts (treatment sample) and 39 non-problematic users (control sample). We used a 2 (participant type: individuals with Internet addiction vs. non-problematic users) × 2 (training type: future event imagination training vs. control condition) × 2 (training session: first session vs. final session) mixed-subjects design to test our hypothesis. The participant type and training type were between the subjects and the training session was within the subject. Half of each sample (the Internet addicts and non-problematic users) was randomly assigned to complete five sessions of future imagination training and the other half was assigned to describe some daily events they had observed. We used the Barratt Impulsivity Scale (BIS) and delay discounting task to assess our outcome variable, such as addiction, impulsivity, and delay discounting rate. The results showed that the future imagination training significantly reduced the delay discounting rate (also for impulsivity and addiction) for both the Internet addicts and non-problematic users than the control condition. Besides, the negative effect of future imagination training on the delay discounting rates (for impulsivity and addiction) remained consistent across the five training sessions. These findings suggest that the future imagination training can be a useful approach to reduce the impulsivity among those who are addicted to the Internet.

## Introduction

One increasingly prominent issue we are facing in the current technological era is Internet addiction (Lai et al., 2013; Wang et al., 2013; Cheng and Li, 2014; Kuss and Lopezfernandez, 2016). There has been no unified standard for the definition of Internet addiction. The most representative one is proposed by Young, who defines the Internet addiction as “a disorder of impulse control without the effect of addictive substances” (Young, 1998). Through the definition of Young (1998), we think that the Internet addiction is a behavioral addiction phenomenon that distinguishes it from other drug addictions. In this study, we believe that all kinds of Internet addiction are a kind of impulsive behavior disorder. Studying the intertemporal choice of the Internet addicts is helpful to find ways to intervene Internet addiction from the behavioral level. Several known treatment methods have not been able to completely prevent the individuals from making impulsive choices, displaying high delay discounting rates, and ignoring long-term negative consequences (Winkler et al., 2013). In fact, the behavior of staying online despite the long-term negative consequences among the Internet addicts is a representation of delay discounting. Delay discounting means that compared with current or recent benefits, people tend to give future benefits less weight, and choose current or recent benefits (Green and Myerson, 2004). Delay discounting occurs when less weight is given to the future rewards or benefits in comparison with the current benefits (Loewenstein, 1988; Frederick et al., 2002). When faced with the choice between a smaller, more immediate reward and a bigger but delayed one, people tend to choose the former rather than the latter.

The previous studies have reported excessive delay discounting rates in individuals with a variety of addictive disorders (MacKillop et al., 2011; Amlung et al., 2017; Wölflinga et al., 2020). A high prevalence of delay discounting has been noted among the individuals with opioid (Madden et al., 1997) or marijuana (Johnson et al., 2010) dependence, and in those with heroin (Kirby et al., 1999), and smoking (Ohmura et al., 2005) addiction. Such high rates of delay discounting have been observed among the individuals battling pathological gambling (Alessi and Petry, 2003) and Internet addiction (Saville et al., 2010; Li et al., 2016a,b, 2019; Weinstein et al., 2016). This notion of high delay discounting rate among the addicts is also supported in research on adolescent substance abuse (Stanger et al., 2012). This line of research has shown that individuals with addictions tend to exhibit a greater willingness to obtain immediate but small benefits at the expense of long-term but considerable ones (e.g., health, familial happiness, and good social relationships).

To better understand the relationship between the delay discounting and addictive behaviors, the researchers have focused their investigations on the neural bases for delay discounting and proposed different theories to explain its occurrence, such as the dual process model (McClure et al., 2004; Li et al., 2019). Peters and Büchel (2011) established a selection mechanism model of addiction that includes valuation, cognitive control, and imagery/prospection networks. Peters and Büchel (2010) noted that delay discounting might decrease after engaging the participants in prospecting or imagining the future. Moreover, the researchers found that impairments in the imagery/prospection network can result in the excessive delay discounting rates (Kwan et al., 2002).

Building on the literature reviewed above, we speculated that the high delay discounting rates of addictive individuals may be caused by their long-term exposure to the drugs or behaviors that affect the imagery/prospection ability. When the imagery/prospection networks are impaired, they may not be able to envision the future very often and therefore, fail to experience the potential benefits of future thinking. Without future thinking, they may end up with the excessive rates of delay discounting. The past studies suggested that expectation is the source of the effects, which, in turn, can increase or decrease the subjective value of future rewards (Loewenstein, 1987). Peters and Büchel (2010) conducted an functional magnetic resonance imaging (fMRI) study and demonstrated that episodic future thinking could reduce the delay discounting rates through the modulation of neural decision-making and episodic future thinking networks when the participants spontaneously engaged in episodic prospection. In addition, Benoit et al. (2011) found that if a participant vividly imagines positive events that occur in the future would decrease their delay discounting rates. It remains unclear whether the delay discounting rate of Internet addicts could be successfully reduced through the long-term training employing future imagination task. The recent studies found that episodic future thinking can decrease the delay discounting rate among the non-addictive individuals (Stein et al., 2016; Hu et al., 2017; Wu et al., 2017; Scholten et al., 2019).

Episodic future thinking refers to the process of thinking and imagining future events based on the current moment or the past experiences (Atance and O'Neill, 2001). Episodic future thinking is to extract the relevant situational information from the original memory of the individual and then project it onto the future. In other words, episodic future thinking aimed to create a future image using the past experiences (Lechner et al., 2019). Episodic future thinking can stimulate individuals to make far-sighted choices, such as planning for the future, making long-term decisions, and achieving long-term goals (Atance and O'Neill, 2001; D'Argembeau et al., 2012). A prior literature has shown that individuals who imagine future scenarios while performing intertemporal choice could significantly reduce the delay discounting rate (Daniel et al., 2015). An fMRI study showed that imaging future events activated the brain regions associated with the imagining future scenarios more than the normal events (D'Argembeau et al., 2010). A research on alcohol (Bulley and Gullo, 2017) and nicotine (Chiou and Wu, 2017) found that episodic future thinking could reduce the need for alcohol and nicotine. In addition, episodic future thinking can also reduce the cravings (Stein et al., 2018).

The individuals who are deficient in intertemporal choice are more likely to make impulsive decisions. Therefore, the formulation of effective interventions has broad implications for the treatment of addiction. The studies have found that changing the delay discount rate of addicted individuals can change their value of the addictive goods or their self-management (McClure et al., 2013; Bernstein et al., 2014). The experimental studies showed that episodic future thinking training can reduce evaluation of imaginary beverages of the addicts (Snider et al., 2016), and improve the self-management of tasty snacks in smokers (Stein et al., 2016), overweight adults, and children (Daniel et al., 2013, 2015; O'Neill et al., 2015).

In the current study, we employed future imagination training to reduce the delay discounting rates among the Internet addicts. We hope to contribute to the theoretical understanding of delay discounting and to demonstrate future imagination training as an effective method for the intervention for Internet addiction.

## Present Study and Hypotheses

The present study aimed to test the effectiveness of future imagination training as an intervention for behavioral addictions. We hypothesized that the delay discounting rates of participants in the future imagination training condition would be significantly lower than those participants in the control condition, regardless of whether they had an Internet addiction. We also expected delay discounting rates in the final session of future imagination training to be significantly lower than those in the first. In addition, we expected impulsivity to be significantly lower among the recipients of future imagination training than among the participants assigned to the control condition. Finally, we assumed that the Internet addiction scores would be significantly lower following the final session of future imagination training than before the experimental training session.

## Materials and Methods

### Experimental Design

The present study employed a 2 (participant type: individuals with Internet addiction vs. non-problematic users) × 2 (training type: future event imagination training vs. control condition) × 2 (training session: first session vs. final session) mixed experimental design. The dependent variables assessed in this study were the delay discounting rates, impulsivity in the first and final sessions of training, and the Internet addiction scores after the experimental training.

### Participants

In this study, 40 individuals (16 women, 24 men; mean age = 19 years) being treated for Internet addiction were recruited from an Internet addiction withdrawal school located in Beijing, China, and 39 non-problematic users (19 women, 20 men; mean age = 19 years), from the BBS campus of Beijing Normal University located in Beijing. The participants rated on a scale ranging from 1 (rarely) to 5 (always) their agreement with the statements in the Internet addiction test (Young et al., 1999). A total score of more than 49 will be diagnosed as an Internet addiction. A total score of below 49 will be defined as non-problematic users in the current study. We have ensured that all the individuals with Internet addiction in this study met the diagnostic criteria for Internet addiction. The individuals with Internet addiction and non-problematic users were well-matched in terms of age and gender. All the participants had never participated in an experiment similar to the present one. The additional characteristics of the participants are presented in Table 1.

**Table 1:** The characteristics of participants (*M* ± *SD*).

**Participant group**	**Training type**	***n***	**Male (%)**	**Female (%)**	**Age**	**Education years**	**Addiction scores**
Non-problematic users	Imagining the Future	20	50	50	19.11 ± 1.79	11.35 ± 0.81	24.90 ± 2.79
	Control	19	52	48	19.26 ± 1.04	11.68 ± 0.75	24.84 ± 3.47
Internet addicts	Imagining the Future	20	60	40	18.80 ± 2.72	11.26 ± 1.10	47.00 ± 8.79
	Control	20	60	40	18.52 ± 1.78	11.00 ± 1.18	47.10 ± 8.84

### Ethics Statement

The present study was reviewed and approved by the Committee of Protection of Subjects at Beijing Normal University. Each participant provided a written informed consent before the study was conducted, and was fully debriefed at the end of the research, in accordance with the guidelines established by the committee. Each participant received a gift at the end of the study.

### Materials

#### Assessments

We assessed the participants' delay discounting (a delay discounting task), impulsivity [Barratt Impulsivity Scale (BIS)], and addiction (Internet Addiction Scale) during the first session and the fifth session of the five-session training.

##### The Internet Addiction Test (IAT)

The Internet addiction test (IAT) is known to be a reliable and valid measure of addictive use of the Internet (Young et al., 1999). The scale consists of 20 items and four factors. The respondents can rate the applicability of items to themselves using 5-point Likert-type scales, with 1 = “rarely,” 2 = “occasionally,” 3 = “frequently,” 4 = “often,” and 5 = “always.” Each item also contains a “not applicable” option. Obtaining a score of 49 or less on the scale indicates that an individual is an average online user who might surf the Web a bit too long at times, but who still has control over his or her usage. A score between 50 and 79, on the other hand, means that an individual experiences occasional or frequent problem due to Internet use. Finally, a score between 80 and 100 denotes a serious addiction to the Internet. The Cronbach's alpha of 0.9085 demonstrates that the Chinese version of the scale possesses good reliability and validity, as shown by Cao et al. (2010).

##### The Barratt Impulsivity Scale (BIS-11)

The 11th edition of BIS scale developed by Patton et al. (1995) was adapted into Chinese by Zhou et al. (2006). The scale, which has 26 items, is comprised of three factors. The individuals respond to the items on the scale by indicating their answers on a 4-point Likert scale, with 1 = “rarely,” 2 = “occasionally,” 3 = “frequently,” and 4 = “always.” The level of impulsivity of an individual is diagnosed by calculating the total score obtained on the scale, with the higher scores signifying greater impulsivity. The test-retest coefficients of the total scale and subscales (attention impulsiveness, motor impulsiveness, and non-planning impulsiveness) have previously been reported as being 0.853, 0.765, 0.791, and 0.838, respectively; the Cronbach's alphas are 0.759, 0.765, 0.658, and 0.687, respectively. The Chinese version thus shows good reliability and validity.

##### The Delay Discounting Task

The delay discounting task used in this study was adopted from the research of Chen and He (2011). The delay discounting was assessed with a paper-and-pencil test containing 19 binary-choice items (with A representing current options and B, “6 months after” future options). The total monetary reward for the future options was fixed at 1,000 RMB (US$158), while that for the current options ranged from a possible 50 (US$7.90) to 950 RMB (US$150.11). The participants could stand to win a set number of 1,000 RMB reward for selecting delayed options (choice B), or a smaller reward (e.g., 50 RMB, 950 RMB) for selecting immediate options (choice A), as outlined below:

“Imagine a situation in which you have a choice for either receiving less money right now, or a larger amount 6 months later. Which would you choose?

1. A: Get 50 RMB (US$7.90) now; B: Wait for 6 months to get 1,000 RMB (US$150.11).
2. A: Get 100 RMB (US$15.80) now; B: Wait for 6 months to get 1,000 (US$150.11) RMB.
3. A: Get 150 RMB (US$23.70) now; B: Wait for 6 months to get 1,000 RMB (US$150.11).
4. A: Get 200 RMB (US$31.60) now; B: Wait for 6 months to get 1,000 RMB (US$150.11).
5. A: Get 250 RMB (US$39.50) now; B: Wait for 6 months to get 1,000 RMB (US$150.11).
6. A: Get 300 RMB (US$47.40) now; B: Wait for 6 months to get 1,000 RMB (US$150.11).
7. A: Get 350 RMB (US$55.30) now; B: Wait for 6 months to get 1,000 RMB (US$150.11).
8. A: Get 400 RMB (US$63.20) now; B: Wait for 6 months to get 1,000 RMB (US$150.11).
9. A: Get 450 RMB (US$71.10) now; B: Wait for 6 months to get 1,000 RMB (US$150.11).
10. A: Get 500 RMB (US$79) now; B: Wait for 6 months to get 1,000 RMB (US$150.11).
11. A: Get 550 RMB (US$86.90) now; B: Wait for 6 months to get 1,000 RMB (US$150.11).
12. A: Get 600 RMB (US$94.80) now; B: Wait for 6 months to get 1,000 RMB (US$150.11).
13. A: Get 650 RMB (US$102.71) now; B: Wait for 6 months to get 1,000 RMB (US$150.11).
14. A: Get 700 RMB (US$110.61) now; B: Wait for 6 months to get 1,000 RMB (US$150.11).
15. A: Get 750 RMB (US$118.71) now; B: Wait for 6 months to get 1,000 RMB (US$150.11).
16. A: Get 800 RMB (US$126.41) now; B: Wait for 6 months to get 1,000 RMB (US$150.11).
17. A: Get 850 RMB (US$134.51) now; B: Wait for 6 months to get 1,000 RMB (US$150.11).
18. A: Get 900 RMB (US$142.21) now; B: Wait for 6 months to get 1,000 RMB (US$150.11).
19. A: Get 950 RMB (US$150.11) now; B: Wait for 6 months to get 1,000 RMB (US$150.11).”

#### Training Program

We followed the prior researchers in designing our future imagination trainings (Levine et al., 2002). The participants were asked to imagine the following six aspects of events so that they could better imagine the future events (Levine et al., 2002). The six aspects were presented on a sheet of paper. The training program aimed to enable participants to imagine future events and to write down these events on a sheet of paper in 15 min. In contrast, those in the control group were asked to imagine occurring events instead in 15 min. All the participants imagined and wrote following the instruction of six aspects of the events. The six aspects are as follows:

1. Event information: what the weather was like at the time of the event, and what other people were wearing.
2. Time information: when the event took place. For example, in the morning or afternoon.
3. Temporal integration of information: the events before and after the event.
4. Location of information: where the event took place.
5. Other sensory information: the hearing, vision, taste, smell, and body intuition of the subject when the event occurs.
6. Emotional information: the emotional and psychological activities of the subjects at the time of the event.

### Procedure

#### Pre-Training Session

The IAT was used to evaluate all the participants in the study. In this session, the participants with Internet addiction were assigned to either the group receiving future imagination training (*n* = 20) or the control group (*n* = 20) according to their gender, and their Internet addiction scores. The non-problematic users were also assigned to one of these conditions according to their gender and Internet addiction score. There were 20 such participants in the experimental training group and 19 in the control group.

#### Training Sessions

In these sessions, the participants with the Internet addiction and non-problematic users were given the requisite training according to their group. Each group completed the training program. The research participants underwent five sessions of training, held at 6-day intervals, within the period of 1 month.

##### Future Imagination Training

The participants first completed a training task in each session of this training program. In the first stage of training, the participants were asked to imagine events occurring 1 week from the present day. After the first training session, each participant was evaluated using the IAT, BIS, and the delay discounting task. In the second stage, they were asked to imagine events occurring after 1 month. In the third, they were asked to imagine events occurring after 1 year, and in the fourth, events occurring after 10 years. In the fifth session, the final stage of training, the participants were asked to imagine events occurring 50 years from the present day. Each session, the participants were also asked to vividly describe the future imagination events and write down on the paper.

##### Control Training

In each session of this training program, they were instructed to write down a description of a present-day event. Each time, they were also asked to vividly describe the present-day events. After the first training session, they were evaluated using the IAT, BIS, and the delay discounting task.

#### Post-Training Session

After the final training session, all the participants were re-evaluated using the IAT, BIS, and the delay discounting task. Furthermore, all the participants were asked to fill out the items addressing the demographic variables at the end of the questionnaire, such as age, gender, and years of education.

## Results

### Participant Characteristics and Group Allocation

#### The Addiction Scores as the Dependent Variable

The addiction scores across the two groups did not vary as a function of participant age [*F*_(1, 75)_ = 3.002, *p* = 0.087, η^2^ = 0.038] and education [*F*_(1, 75)_ = 3.038, *p* = 0.085, η^2^ = 0.039]. However, there was a significant difference in the addiction scores across the two subject groups (addicts vs. non-addicts), *F*_(1, 75)_ = 219.165, *p* < 0.001, η^2^ = 0.745. These results suggested that the two groups of subjects in the current study were comparable across age and education.

### Dependent Variables Analysis

In the current study, we aimed to perform a descriptive statistical analysis of the results. So, we used SPSS Version 16.0 (IBM Corp., NY, USA) to analyze our experimental data. In the result part, we used the statistical method of ANOVA. A 2 (participant type: individuals with Internet addiction vs. non-problematic users) × 2 (training type: future event imagination training vs. control condition) × 2 (training session: first session vs. final session) ANOVA analysis was conducted for each of the two dependent variables of delay discounting rate and impulsivity. The descriptive statistics of the different participant groups in the imagining the future and control conditions are shown in Table 2, which reveal decreases in the delay discounting rates and impulsivity.

**Table 2:** Different participant groups in the experimental and control conditions showing decreases in the delay discounting rates and impulsivity (*M* ± *SD*).

**Participant group**	**Training type**	***n***	**Delay discounting rate**		**Impulsivity**	
			**First session**	**Final session**	**First session**	**Final session**
Non-problematic users	Imagining the future	20	1.30 ± 0.38	1.11 ± 0.23	52.00 ± 5.24	47.90 ± 6.50
	Control	19	1.36 ± 0.43	1.41 ± 0.43	52.84 ± 7.23	54.26 ± 13.58
Internet addicts	Imagining the future	20	1.55 ± 0.50	1.22 ± 0.31	61.68 ± 10.41	52.21 ± 7.15
	Control	20	1.78 ± 3.96	1.83 ± 0.44	58.86 ± 6.42	58.14 ± 8.15

#### The Delay Discounting Rate as the Dependent Variable

**Figure 1:**
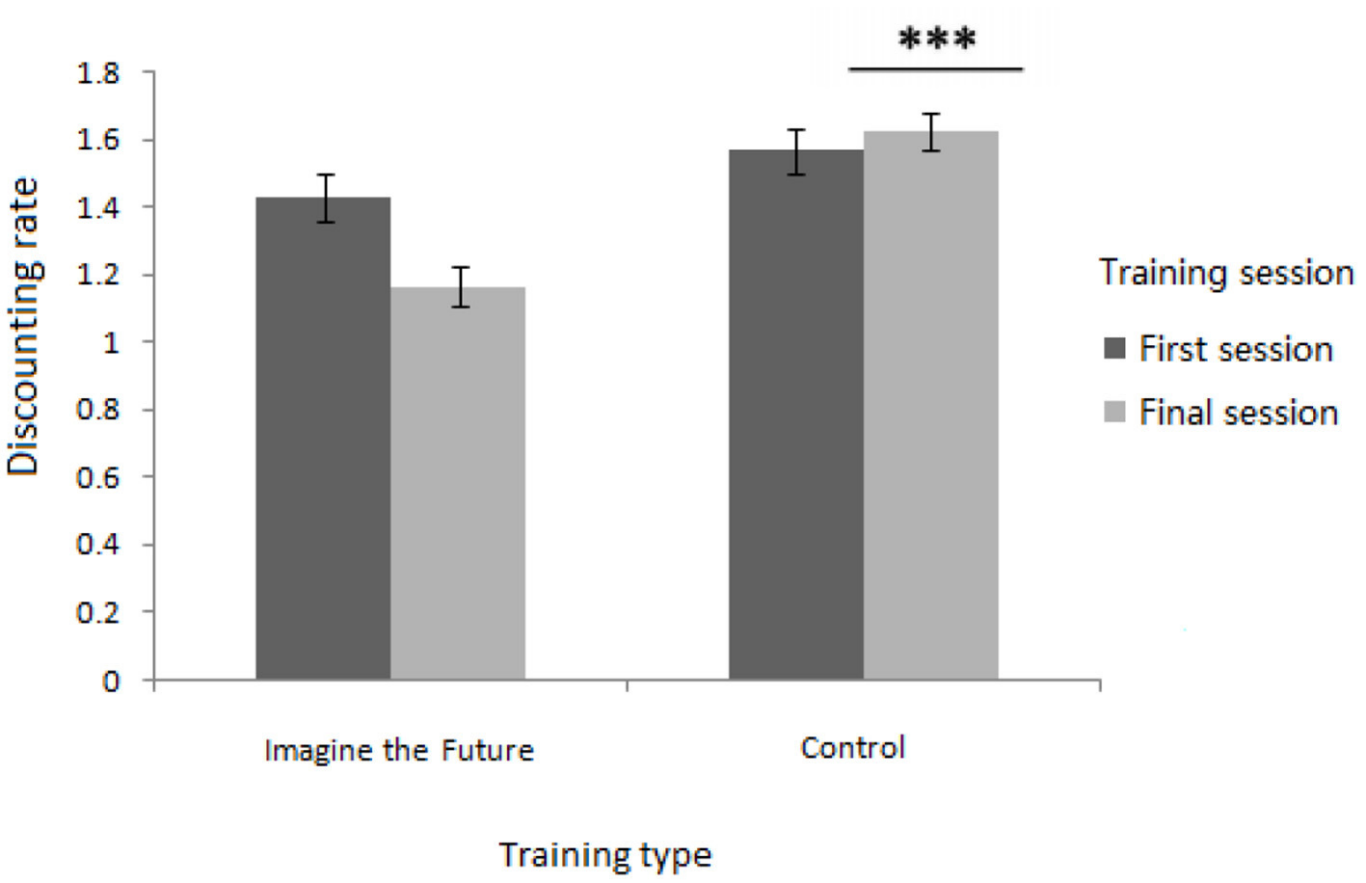
Interaction between the training session and training type for the delay discounting rates (includes mean and SD; ^***^means *p* < 0.001).

The results revealed a significant main effect of subject [*F*_(1, 75)_ = 13.881, *p* < 0.001]. The delay discounting rates of individuals with the Internet addiction (*M* = 1.594, *SD* = 0.056) were significantly higher than those of the non-problematic users (*M* = 1.296, *SD* = 0.056). A significant main effect of training type was also found [*F*_(1, 75)_ = 25.920, *p* < 0.001, η^2^ = 0.29], and the delay discounting rates of participants who underwent future imagination training (*M* = 1.295, *SD* = 0.057) were significantly lower than those in the control condition (*M* = 1.595, *SD* = 0.056). The analysis also showed a significant interaction between the training session and training type [*F*_(1, 75)_ = 15.670, *p* < 0.001, η^2^ = 0.173] (as shown in Figure 1). With regard to the delay discounting rates of the participants recorded in the final training session, a simple effect analysis demonstrated that there was a significant difference by training type [*F*_(1, 77)_ = 9.240, *p* < 0.001]. The delay discounting rates of participants in the experimental training condition (*M* = 1.163, *SD* = 0.274) were significantly lower than those of participants in the control condition (*M* = 1.634, *SD* = 0.482). As for the delay discounting rates of participants in the first training session, a simple effect analysis demonstrated that there was no significant difference by training type [*F*_(1, 77)_ = 0.417, *p* = 0.521]. These results indicated that the future imagination training can reduce the delay discounting rates of an individual.

#### Impulsivity as the Dependent Variable

**Figure 2:**
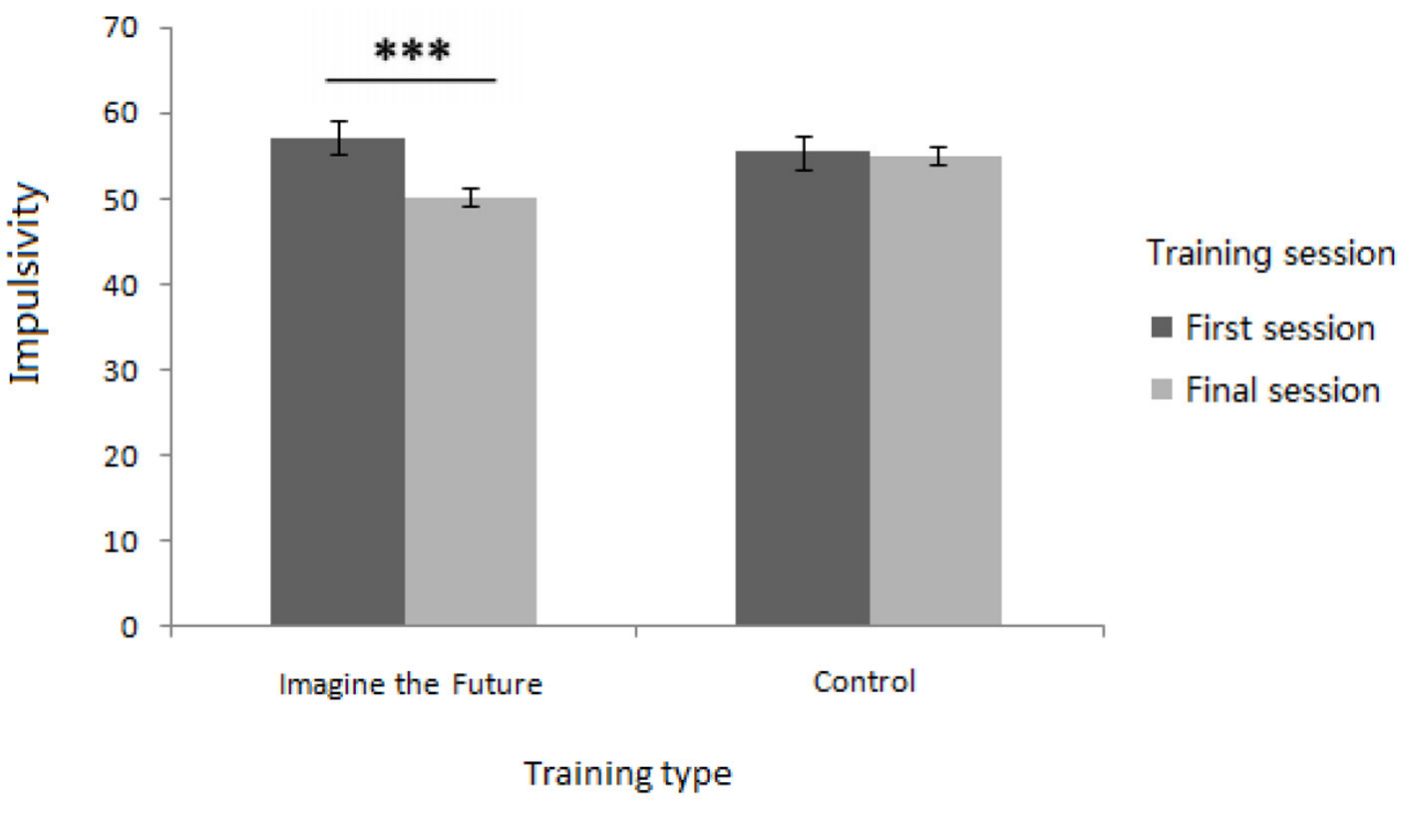
Interaction between the training session and training type for impulsivity scores (includes mean and SD; ^***^means *p* < 0.001).

In the current study, we aimed to detect whether future imagination training could reduce the impulsivity scores of the individuals. A significant main effect of subject was detected [*F*_(1, 75)_ = 20.553, *p* < 0.001, η^2^ = 0.215]. The impulsivity scores of individuals with Internet addiction (*M* = 57.724, *SD* = 1.029) were significantly higher than those of non-problematic users (*M* = 51.084, *SD* = 1.042). A significant main effect of first/final training session was also found [*F*_(1, 75)_ = 19.505, *p* < 0.001, η^2^ = 0.206], and the impulsivity scores of participants in the final session (*M* = 52.557, *SD* = 8.377) were significantly lower than those in the first session (*M* = 56.354, *SD* = 8.416). A significant interaction between the training session and training type was found [*F*_(1, 75)_ = 13.971, *p* < 0.001, η^2^ = 0.157] (as shown in Figure 2). With regard to the impulsivity scores of participants recorded in the imagining the future training condition, a simple effect analysis demonstrated that there was a significant difference between the first and final sessions [*F*_(1, 77)_ = 31.70, *p* < 0.001]. The impulsivity scores in the final session (M = 50.000, SD = 7.079) were significantly lower than those in the first session (M = 55.050, SD = 8.866). As for the impulsivity scores of participants in the control condition, a simple effect analysis demonstrated that there was no significant difference between the first and final sessions [*F*_(1, 77)_ = 0.720, *p* = 0.432]. There was no significant main effect of training type [*F*_(1, 75)_ = 1.135, *p* = 0.290, η^2^ = 0.015]. These results indicate that the future imagination training can decrease the impulsivity scores of an individual.

#### Internet Addiction Degree as Dependent Variable

**Figure 3:**
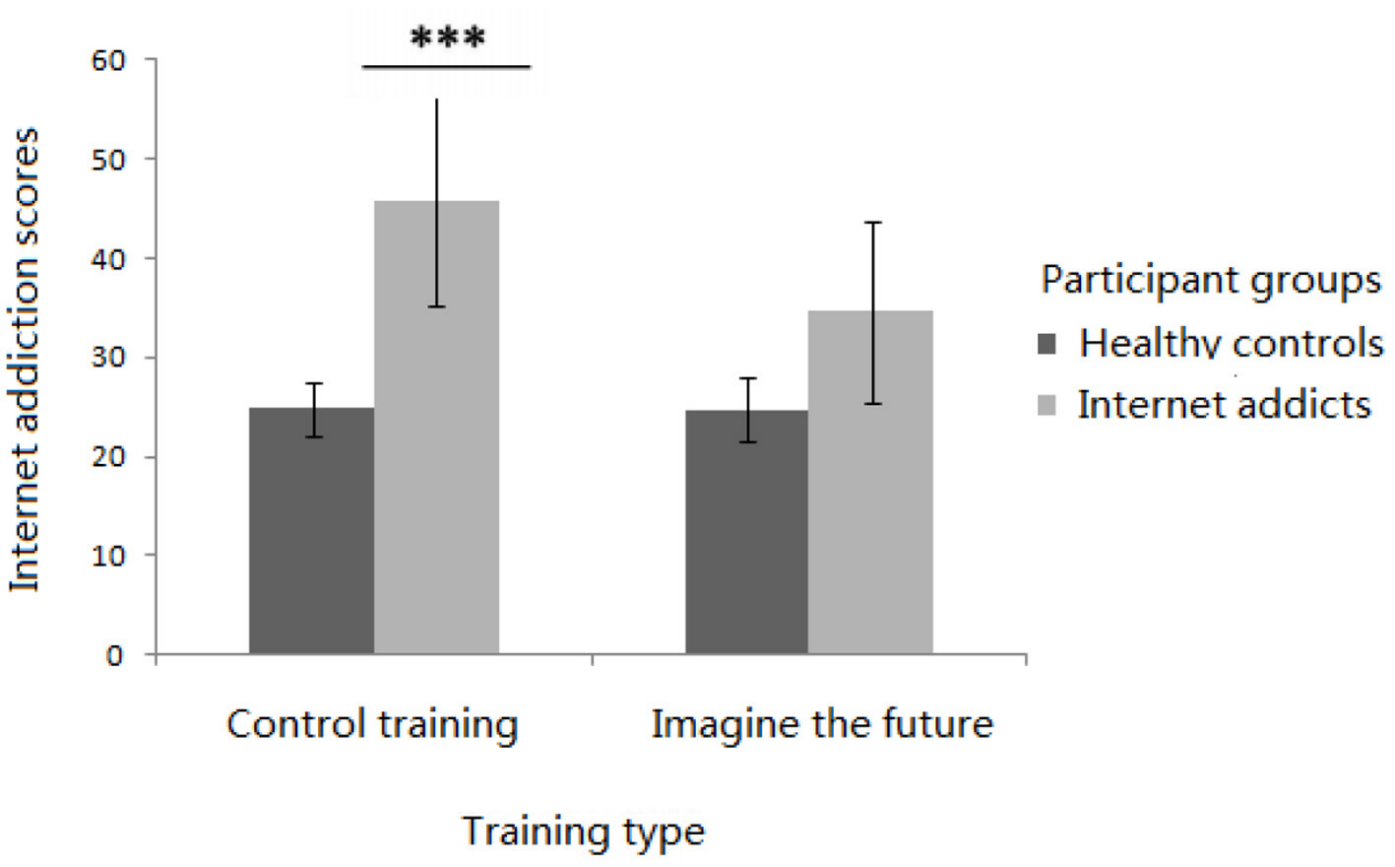
Interaction between the participant groups and training type for Internet addiction scores (includes mean and SD; ^***^means *p* < 0.001).

In the current study, we aimed to assess the effectiveness of imagining the future training among the participants with Internet addiction. We controlled the Internet addiction scores as a covariate in the first sessions of training and used these scores as the dependent variable. The results showed a significant interaction between the training type and participant group [*F*_(1, 76)_ = 18.463, *p* < 0.001, η^2^ = 0.200] (as shown in Figure 3). As for the Internet addiction scores of individual addicts, a simple effect analysis demonstrated that there was a significant main effect of training type [*F*_(1, 37)_ = 36.959, *p* < 0.001, η^2^ = 0.500]. The Internet addiction scores in the imagine the future condition (*M* = 34.677, *SD* = 1.367) were significantly lower than those in the control condition (*M* = 46.149, *SD* = 1.301). As for the healthy control participants, a simple effect analysis demonstrated that there was no significant main effect of training type [*F*_(1, 77)_ = 0.620, *p* = 0.480]. These results indicate that the future imagination training can reduce the degree of Internet addiction of an individual, supporting the effectiveness of future imagination training as an intervention for Internet addiction.

## Discussion

The results of our study confirmed that the future imagination training can decrease the delay discounting rates and is an effective intervention for Internet addiction. The results showed that the delay discounting rates of participants with the Internet addiction and non-problematic users were significantly lower in the future imagination training condition than those in the control condition. We also found that the delay discounting rates, impulsivity, and Internet addiction scores significantly decreased from the first to the final training sessions in the experimental training condition but remained unchanged in the control condition. Overall, these results supported our hypothesis and demonstrated that future imagination training can be an effective intervention for the behavioral addictions.

These results concur with the previous research showing that episodic future thinking could reduce the delay discounting rates in the Internet addicts and non-problematic users (Peters and Büchel, 2010; Benoit et al., 2011; Daniel et al., 2015; Dassen et al., 2016; Stein et al., 2016; Hu et al., 2017; Wu et al., 2017; Scholten et al., 2019). The present study offered evidence for the link between the imagination of future and delay discounting rates of the individuals, which is consistent with fMRI research finding that episodic future thinking can reduce the delay discounting rates (Benoit et al., 2011). The fMRI study provided the physiological evidence for the activation of specific brain regions associated with imagining future scenarios (D'Argembeau et al., 2010). In addition, the findings from other types of subjects also showed that the episodic future thinking can reduce the delay discounting rates, such as alcohol addict (Bulley and Gullo, 2017), nicotine addict (Chiou and Wu, 2017), and smoker (Stein et al., 2018).

The current findings show that episodic future thinking is an effective method to reduce the delay discounting rate of an individual. Episodic future thinking enables individuals to make long-term decisions and guide their future related behaviors (Atance and O'Neill, 2001; D'Argembeau et al., 2012). One possible mechanism is that the expectation, as a source of the effects, can increase or decrease the subjective value of future rewards (Loewenstein, 1987). Consequently, the individuals may demonstrate varying (i.e., high or low) rates of delay discounting in the decision-making tasks involving money, as the previous research on the behavioral economics has affirmed (Loewenstein, 1987). The future imagination training can help guide one's life and enable individuals to make rational decisions employing the long-term planning based on their goals. We argue that the training can strengthen the ability of individuals to imagine the future and thus increase or decrease the utility of goods. Based on the dual process model theory (McClure et al., 2004), we speculate that the episodic future thinking can help individuals to resist the current temptation and make a long-term rational decision.

It is worth noting that the current results found that episodic future thinking reduced Internet addiction. A prior literature has found that the episodic future thinking can decrease the degree of the addiction (Daniel et al., 2013, 2015; O'Neill et al., 2015; Stein et al., 2016, 2018; Bulley and Gullo, 2017; Chiou and Wu, 2017). Furthermore, our current results were supported by the previous studies using episodic future thinking to reduce the delay discount rate of addicts and the evaluation of imaginary beverages (Snider et al., 2016). This can be explained by the account that the decreasing of delay discount rate among the addicted individuals may reduce their subjective value of the addictive goods (McClure et al., 2013; Bernstein et al., 2014). Moreover, the episodic future thinking can improve self-management of the Internet addicts, which was supported by the prior research (Stein et al., 2016). The present study provides a viable solution for the lack of effective interventions for the Internet addiction as highlighted by Winkler et al. (2013).

The present study lends further support to the neural mechanism theory of imagery/prospection network. We acquired evidence from the behavioral experiments, which extend our understanding of addictive behavior disorders associated with the high delay discounting rates. The prior research has shown that anticipating the future can increase the self-control (Kirby and Guastello, 2001). There is a possibility that the future imagination training conducted in this study also promoted the self-control abilities of participants and thus decreased their delay discounting rates.

## Limitations and Directions for Future Research

Although this was the first study to investigate how future imagination training may decrease the delay discounting rate among the Internet addicts and non-problematic users, there are still several limitations. Hence, the current findings may suggest several key directions for future research. First, we used BIS to measure the impulsivity of the subjects in this study, and the results showed that the impulsivity of the subjects changed after the end of the experiment. However, the BIS is a trait measure. Given this, we want to conduct further long-term follow-up studies in future to verify the current findings. Second, the current research has only investigated the effect of future imagination training on the intertemporal decision-making, but the psychological mechanism has not been explored. Therefore, in future studies, we plan to conduct the experimental studies on the psychological mechanism by which future imagination can reduce the intertemporal decision-making. Third, the previous studies have concluded that the different manner with which issues are framed can affect the selections of individuals (De Martino et al., 2006) and that emotional valence modulates the delay discounting (Liu et al., 2013). However, we did not consider the effect of different descriptive frameworks on the results. So, we look forward to possibly adding the framing effect to our investigation of the delay discounting rates in the future, through the incorporation of delay discounting tasks requiring participants to frame an issue/event in a variety of ways. It would also be useful to test the emotional intensity of the individuals in the two different frames, and investigate emotional intensity as a moderator between the framing effect and delay discounting tasks. Fourth, the current study only used the self-report and behavioral data. Therefore, further studies could benefit from the neurophysiological or psychophysiological measures integration. Last, although the future imagination training reduced the scores of Internet addicts, the scores of Internet addicts were significantly higher than those of normal Internet users in the current study. So, the future research may consider using longitudinal design to further explore whether the future imagination training can exert a lasting influence on the Internet addicts and even reduce the addictive tendency to be as low as normal subjects.

In sum, the future imagination training can be effectively applied to decrease delay discounting in both the Internet addicts and non-problematic users. This training method may have implications for preventing and reducing the Internet addiction.

## Data Availability Statement

The original contributions presented in the study are included in the article/supplementary material, further inquiries can be directed to the corresponding author.

## Ethics Statement

The studies involving human participants were reviewed and approved by the Committee of Protection of Subjects at Beijing Normal University. The participants provided their written informed consent to participate in the study.

## Author Contributions

HL completed the experiment design, data collection, and article writing.

## Funding

This research was supported by the Research Fund of Capital University of Economics and Business (Codes: XRZ2021031).

## Conflict of Interest

The author declares that the research was conducted in the absence of any commercial or financial relationships that could be construed as a potential conflict of interest.

## Publisher's Note

All claims expressed in this article are solely those of the authors and do not necessarily represent those of their affiliated organizations, or those of the publisher, the editors and the reviewers. Any product that may be evaluated in this article, or claim that may be made by its manufacturer, is not guaranteed or endorsed by the publisher.

## References

[B1] Alessi S. M. Petry N. M. (2003). Pathological gambling severity is associated with impulsivity in a delay discounting procedure. Behav. Proces. 64, 345–354. 10.1016/S0376-6357(03)00150-5 14580703

[B2] Amlung M. Vedelago L. Acker J. Balodis I. M. Mackillop J. (2017). Steep delay discounting and addictive behavior: a meta-analysis of continuous associations. Addiction 112, 51–62. 10.1111/add.13535 27450931 PMC5148639

[B3] Atance C. M. O'Neill D. K. (2001). Episodic future thinking. Trends Cogn. Sci. 5, 533–539. 10.1016/S1364-6613(00)01804-0 11728911

[B4] Benoit R. G. Gilbert S. J. Burgess P. W. (2011). A neural mechanism mediating the impact of episodic prospection on farsighted decisions. J. Neurosci. 31, 6771–6779. 10.1523/JNEUROSCI.6559-10.2011 21543607 PMC6632845

[B5] Bernstein M. Murphy J. MacKillop J. Colby S. (2014). The dose effects of short-term dronabinol (oral THC) maintenance in daily cannabis users. Drug Alcohol Depend. 128, 64–70. 10.1016/j.drugalcdep.2012.08.001 22921474 PMC3546149

[B6] Bulley A. Gullo M. J. (2017). The influence of episodic foresight on delay discounting and demand for alcohol. Addict. Behav. 66, 1–6. 10.1016/j.addbeh.2016.11.003 27837662

[B7] Cao J. Q. Yang J. W. Yang J. (2010). Reliability and validity of internet addiction impairment indexes. Chin. Gen. Pract. 34: 33.

[B8] Chen H. X. He G. B. (2011). The effect of construal level on intertemporal choice and risky choice. Acta Psychol. Sin. 4:11. 10.3724/SP.J.1041.2011.00442

[B9] Cheng C. Li A. Y. (2014). Internet addiction prevalence and quality of (real) life: a meta-analysis of 31 nations across seven world regions. Cyberpsychol. Behav. Soc. Netw. 17, 755–760. 10.1089/cyber.2014.0317 25489876 PMC4267764

[B10] Chiou W. B. Wu W. H. (2017). Episodic future thinking involving the nonsmoking self can induce lower discounting and cigarette consumption. J. Stud. Alcohol Drugs 78, 106–112. 10.15288/jsad.2017.78.106 27936370

[B11] Daniel T. O. Said M. Stanton C. M. Epstein L. H. (2015). Episodic future thinking reduces delay discounting and energy intake in children. Eat. Behav. 18, 20–24. 10.1016/j.eatbeh.2015.03.006 25863227 PMC6504176

[B12] Daniel T. O. Stanton C. M. Epstein L. H. (2013). The future is now: reducing impulsivity and energy intake using episodic future thinking. Psychol. Sci. 24, 2339–2342. 10.1177/0956797613488780 24022653 PMC4049444

[B13] D'Argembeau A. Lardi C. Van der Linden M. (2012). Self-defining future projections: exploring the identity function of thinking about the future. Memory 20, 110–120. 10.1080/09658211.2011.647697 22292616

[B14] D'Argembeau A. Stawarczyk D. Majerus S. Collette F. Van der Linden M. Feyers D.. (2010). The neural basis of personal goal processing when envisioning future events. J. Cogn. Neurosci. 22, 1701–1713. 10.1162/jocn.2009.21314 19642887

[B15] Dassen F. C. M. Jansen A. Nederkoorn C. Houben K. (2016). Focus on the future: episodic future thinking reduces discount rate and snacking. Appetite 96, 327–332. 10.1016/j.appet.2015.09.032 26431684

[B16] De Martino B. Kumaran D. Seymour B. Dolan R. J. (2006). Frames, biases, and rational decision-making in the human brain. Science 313, 684–687. 10.1126/science.1128356 16888142 PMC2631940

[B17] Frederick S. Loewenstein G. O'donoghue T. (2002). Time discounting and time preference: a critical review. J. Econ. Literat. 40, 351–401. 10.1257/jel.40.2.35126063653

[B18] Green L. Myerson J. (2004). A discounting framework for choice with delayed and probabilistic rewards. Psychol. Bullet. 130, 769–792. 10.1037/0033-2909.130.5.769 15367080 PMC1382186

[B19] Hu X. Kleinschmidt H. Martin J. A. Han Y. Thelen M. Meiberth D.. (2017). A reduction in delay discounting by using episodic future imagination and the association with episodic memory capacity. Front. Hum. Neurosci. 10, 1–20. 10.3389/fnhum.2016.00663 28105009 PMC5214699

[B20] Johnson M. W. Bickel W. K. Baker F. Moore B. A. Badger G. J. Budney A. J. (2010). Delay discounting in current and former marijuana-dependent individuals. Exp. Clin. Psychopharmacol. 18:99. 10.1037/a0018333 20158299 PMC2874198

[B21] Kirby K. N. Guastello B. (2001). Making choices in anticipation of similar future choices can increase self-control. J. Exp. Psychol. Appl. 7:154. 10.1037/1076-898X.7.2.154 11477982

[B22] Kirby K. N. Petry N. M. Bickel W. K. (1999). Heroin addicts have higher discount rates for delayed rewards than non-drug-using controls. J. Exp. Psychol. Gen. 128:78. 10.1037/0096-3445.128.1.78 10100392

[B23] Kuss D. J. Lopezfernandez O. (2016). Internet addiction and problematic Internet use: a systematic review of clinical research. World J. Psychiatr. 6, 143–176. 10.5498/wjp.v6.i1.143 27014605 PMC4804263

[B24] Kwan D. Craver C. F. Green L. Myerson J. Rosenbaum R. S. (2002). Dissociations in future thinking following hippocampal damage: evidence from discounting and time perspective in episodic amnesia. J. Exp. Psychol. Gen. 142, 1355–1369. 10.1037/a0034001 23978187

[B25] Lai C. M. Mak K. K. Watanabe H. Ang R. P. Pang J. S. Ho R. C. (2013). Psychometric properties of the Internet Addiction Test in Chinese adolescents. J. Pediatr. Psychol. 38, 794–807. 10.1093/jpepsy/jst022 23671059

[B26] Lechner W. V. Sidhu N. K. Kittaneh A. Anand A. (2019). Interventions with potential to target executive function deficits in addiction: current state of the literature. Curr. Opin. Psychol. 30, 24–28. 10.1016/j.copsyc.2019.01.017 30797130

[B27] Levine B. Svoboda E. Hay J. F. Winocur G. Moscovitch M. (2002). Aging and autobiographical memory: dissociating episodic from semantic retrieval. Psychol. Aging 17:677. 10.1037/0882-7974.17.4.677 12507363

[B28] Li H. Jin S. Guo Y. (2016a). How do construal levels affect the intertemporal choices of Internet addicts? Comput. Hum. Behav. 6060, 173–117. 10.1016/j.chb.2016.02.016

[B29] Li H. X. Guo Y. F. Yu Q. L. (2019). The psychological mechanism of dual processing in intertemporal choice among Internet Addicts and Healthy Controls—the meditational role of self control. Comput. Hum. Behav. 101, 95–103. 10.1016/j.chb.2019.07.010

[B30] Li Q. Tian M. Taxer J. L. Zheng Y. Wu H. Sun S.. (2016b). Problematic internet users' discounting behaviors reflect an inability to delay gratification, not risk taking. Cyberpsychol. Behav. Soc. Netw. 19, 172–178. 10.1089/cyber.2015.0295 26894438

[B31] Liu L. Feng T. Chen J. Li H. (2013). The value of emotion: how does episodic prospection modulate delay discounting? PLoS ONE 8:e81717. 10.1371/journal.pone.0081717 24312341 PMC3842935

[B32] Loewenstein G. (1987). Anticipation and the valuation of delayed consumption. Econ. J. 666–684. 10.2307/2232929

[B33] Loewenstein G. F. (1988). Frames of mind in intertemporal choice. Manag. Sci. 34, 200–214. 10.1287/mnsc.34.2.200

[B34] MacKillop J. Amlung M. T. Few L. R. Ray L. A. Sweet L. H. Munafò M. R. (2011). Delayed reward discounting and addictive behavior: a meta-analysis. Psychopharmacology 216, 305–321. 10.1007/s00213-011-2229-0 21373791 PMC3201846

[B35] Madden G. J. Petry N. M. Badger G. J. Bickel W. K. (1997). Impulsive and self-control choices in opioid-dependent patients and non-drug-using control patients: drug and monetary rewards. Exp. Clin. Psychopharmacol. 5:256. 10.1037/1064-1297.5.3.256 9260073

[B36] McClure E. A. Vandrey R. G. Johnson M. W. Stitzer M. L. (2013). Effects of varenicline on abstinence and smoking reward following a programmed lapse. Nicotine Tobacco Res. 15, 139–148. 10.1093/ntr/nts101 22573730 PMC3524062

[B37] McClure S. M. Laibson D. I. Loewenstein G. Cohen J. D. (2004). Separate neural systems value immediate and delayed monetary rewards. Science 306, 503–507. 10.1126/science.1100907 15486304

[B38] Ohmura Y. Takahashi T. Kitamura N. (2005). Discounting delayed and probabilistic monetary gains and losses by smokers of cigarettes. Psychopharmacology 182, 508–515. 10.1007/s00213-005-0110-8 16167142

[B39] O'Neill J. Daniel T. O. Epstein L. H. (2015). Episodic future thinking reduces eating in a food court. Eat. Behav. 20, 9–13. 10.1016/j.eatbeh.2015.10.002 26562686 PMC9188833

[B40] Patton J. H. Stanford M. S. Barratt E. S. (1995). Factor structure of the Barratt impulsiveness scale. J. Clin. Psychol. 51, 768–774. 10.1002/1097-4679(199511)51:6<768::AID-JCLP2270510607>3.0.CO;2-1 8778124

[B41] Peters J. Büchel C. (2010). Episodic future thinking reduces reward delay discounting through an enhancement of prefrontal-mediotemporal interactions. Neuron 66, 138–148. 10.1016/j.neuron.2010.03.026 20399735

[B42] Peters J. Büchel C. (2011). The neural mechanisms of inter-temporal decision-making: understanding variability. Trends Cogn. Sci. 15, 227–239. 10.1016/j.tics.2011.03.002 21497544

[B43] Saville B. K. Gisbert A. Kopp J. P. Telesco C. (2010). Internet addiction and delay discounting in college students. Psychol. Rec. 60, 273–286. 10.1007/BF03395707

[B44] Scholten H. Scheres A. de Water E. Graf U. Granic I. Luijten M. (2019). Behavioral trainings and manipulations to reduce delay discounting: a systematic review. Psychon. Bullet. Rev. 26, 1803–1849. 10.3758/s13423-019-01629-2 31270766 PMC6863952

[B45] Snider S. E. LaConte S. M. Bickel W. K. (2016). Episodic future thinking: expansion of the temporal window in individuals with alcohol dependence. Alcohol. Clin. Exp. Res. 40, 1558–1566. 10.1111/acer.13112 27246691 PMC5497459

[B46] Stanger C. Ryan S. R. Fu H. Landes R. D. Jones B. A. Bickel W. K.. (2012). Delay discounting predicts adolescent substance abuse treatment outcome. Exp. Clin. Psychopharmacol. 20:205. 10.1037/a0026543 22182419 PMC3906638

[B47] Stein J. S. Tegge A. N. Turner J. K. Bickel W. K. (2018). Episodic future thinking reduces delay discounting and cigarette demand: an investigation of the good-subject effect. J. Behav. Med. 41, 269–276. 10.1007/s10865-017-9908-1 29270887

[B48] Stein J. S. Wilson A. G. Koffarnus M. N. Daniel T. O. Epstein L. H. Bickel W. K. (2016). Unstuck in time: episodic future thinking reduces delay discounting and cigarette smoking. Psychopharmacology 233, 3771–3778. 10.1007/s00213-016-4410-y 27553824 PMC9812225

[B49] Wang L. Luo J. Bai Y. Kong J. Luo J. Gao W.. (2013). Internet addiction of adolescents in China: prevalence, predictors, and association with well-being. Addict. Res. Theor. 21, 62–69. 10.3109/16066359.2012.690053

[B50] Weinstein A. Abu H. B. Timor A. Mama Y. (2016). Delay discounting, risk-taking, and rejection sensitivity among individuals with Internet and Video Gaming Disorders. J. Behav. Addict. 5, 674–682. 10.1556/2006.5.2016.081 27958761 PMC5370373

[B51] Winkler A. Dörsing B. Rief W. Shen Y. Glombiewski J. A. (2013). Treatment of Internet addiction: a meta-analysis. Clin. Psychol. Rev. 33, 317–329. 10.1016/j.cpr.2012.12.005 23354007

[B52] Wölflinga K. Duvenb E. Wejberac M. Beutela M. E. Müller K. W. (2020). Discounting delayed monetary rewards and decision making in behavioral addictions – a comparison between patients with gambling disorder and internet gaming disorder. Addict. Behav. 108:106446. 10.1016/j.addbeh.2020.106446 32408114

[B53] Wu W.-H. Cheng W. Chiou W.-B. (2017). Episodic future thinking about the ideal self induces lower discounting, leading to a decreased tendency toward cheating. Front. Psychol. 8:287. 10.3389/fpsyg.2017.00287 28303111 PMC5332433

[B54] Young K. Pistner M. O'mara J. Buchanan J. (1999). Cyber disorders: the mental health concern for the new millennium. CyberPsychol. Behav. 2, 475–479. 10.1089/cpb.1999.2.475 19178220

[B55] Young K. S. (1998). Internet addiction: the emergence of a new clinical disorder. CyberPsychol. Behav. 1, 237–244. 10.1089/cpb.1998.1.237

[B56] Zhou L. Xiao S. Y. He X. Y. Li J. Liu H. M. (2006). Reliability and validity of Barratt Impulsiveness Scale-11. Chin. J. Clin. Psychol. 14, 343–344.

